# Investigation of Phase Transformation of Fe65Ni35 Alloy by the Modified Pulse Method

**DOI:** 10.3390/ma13153425

**Published:** 2020-08-03

**Authors:** Janusz Terpiłowski, Stanisław Jóźwiak, Rafał Rudzki, Robert Szczepaniak, Grzegorz Woroniak

**Affiliations:** 1 Institute of Aviation Technology, Faculty of Mechatronics and Aerospace, Military University of Technology, Kaliskiego 2, 00-908 Warsaw, Poland; 2 Institute of Materials Science and Engineering, Faculty of Advanced Technologies and Chemistry, Military University of Technology, Kaliskiego 2, 00-908 Warsaw, Poland; stanislaw.jozwiak@wat.edu.pl; 3 Lukasiewicz Research Network–Institute of Aviation, Al. Krakowska 110/114, 02-256 Warsaw, Poland; rudrav@o2.pl; 4 Aeronautics Faculty, Military University of Aviation, Dywizjonu 303 Str., 08-521 Deblin, Poland; r.szczepaniak@law.mil.pl; 5 HVAC Department, Bialystok University of Technology, Wiejska 45E, 15-351 Bialystok, Poland; g.woroniak@pb.edu.pl

**Keywords:** thermal diffusivity, modified-pulse method (MPM), iron–nickel alloys, phase transitions, flash method

## Abstract

This paper presents the possibility of using a modified-pulse method (MPM) determining the temperature characteristics of thermal diffusivity in order to identify phase transformations in metals. The experiment and attempt of phase identification were conducted for the Fe65Ni35 alloy in the 20–500 °C temperature range during both sample heating and cooling. The estimated error of discrete thermal diffusivity measurements was less than 3%. The method allows us to narrow down the averaging of the interval of this value, as a function of temperature, in the range below 1 K. Recently published analysis of the phase diagrams of Fe–Ni alloys, and the results of the authors’ own research into the Fe65Ni35 alloy, showed very good correlation between changes occurring when heating the alloy and the equilibrium diagram provided by Cacciamani G., Dinsdale A., Palumbo M., and Pasturel A. (Intermetallics 18, 2010, 1148–1162) showing the position of phases with a crystal-lattice structure based on the face-centered cubic (FCC) cell.

## 1. Introduction

Investigations of the thermal properties of alloys or materials, such as thermal diffusivity, conductivity, or expansion play a very important role in today’s world. Interesting investigations on the thermal properties of an invar-type material were conducted by Yichun Liu et al. [1]. Wang et al. [2] investigated the thermal properties of ceramic thermal-barrier coatings using the thermal-conductivity parameter to assess the suitability of the materials for technological applications. Thermal diffusivity is an important thermodynamic property because it is suitable for predicting material behavior in many heat-transfer applications. Reza et al. [3] used this parameter in deuterium-implanted tungsten investigations. Bellucci et al. [4] used thermal diffusivity in research on graphene nanoplatelets using the pulse method. Our work shows how to assess the thermodynamic properties of a material using the thermal diffusivity with a modified-pulse method (MPM).

The use of this method, which is significantly different from other methods used to determine the thermal diffusivity of solids [5,6,7,8,9,10,11,12,13,14,15,16,17,18,19], was justified, above all, by its much higher accuracy in determining the *a*(*T*) of the tested materials. a(T) characteristics are created using a set of discrete thermal-diffusivity values ${a\left(T_{i}\right)|}_{T_{i}-0.5\Delta T}^{T_{i}+0.5\Delta T}$. In the case of the MPM, the estimated measurement error of this value was below 3%, and temperature-averaging range ∆*T* could be narrowed to values less than 1 K. On the other hand, the basic bibliography regarding phase transformations occurring in Fe–Ni alloys is made up of review works [20,21,22,23,24,25,26,27,28,29,30], and studies into the invar temperature range [31,32,33,34,35,36,37,38].

Our first work in this area [39] concerned the study and interpretation of the temperature characteristics *a*(*T*) of the Fe61Ni39, Fe52Ni48, and Fe40Ni60 alloys in the temperature range of 20–700 °C. The next work [40] concerned the interpretation of characteristics a(T) of the metastable Fe80Ni20 alloy, ranging from ambient temperature to about 650 °C. This work concerns the interpretation of thermal-diffusivity characteristics a(T) of the Fe65Ni35 alloy. The flash or pulse method can be used interchangeably, they refer to the same method of creating a surface heat source on the sample front side.

## 2. Short Description of Method and Test Bench

The MPM for the measurement of thermal diffusivity was previously described in detail in [41,42]. The determination of thermal diffusivity by the MPM means is based on the theoretical determination of the temperature distribution inside an opaque and adiabatic sample, and the difference in temperature between two lateral surfaces after the laser pulse is fired on the front surface. In this case, a one-dimensional model was assumed that approximated the actual heat exchange in the sample-environment system. The next step in the research was to record a temporary temperature difference between the front and back surfaces of the sample, resulting in a one-dimensional temperature equalization process in the sample. Lastly, it was estimated how to best match the results of the experiment with a curve, obtaining them as one of several theoretical curves that solve the problem. The optimization parameter was thermal diffusivity, and the value corresponding to the best fit is considered as accurate.

The theoretical temperature distribution $\Theta \left(x,t\right)=T\left(x,t\right)-T_{0}$ in an adiabatic sample, where *T*_0_—thermostating temperature of the sample:

(1) $${\left(\frac{\vartheta \Theta }{\vartheta x}\right)}_{x=0}={\left(\frac{\vartheta \Theta }{\vartheta x}\right)}_{x=l}=0$$

when the temperature inside the sample at time t=0 is equal to Θ(x, 0), according to [43], it is given by Equation (2); when the surface layer (0 ≤ x ≤ g ≪ l) absorbs the radiation-pulse energy of surface density Q, with respect to initial conditions, the theoretical temperature distribution is given by Equation (3): 
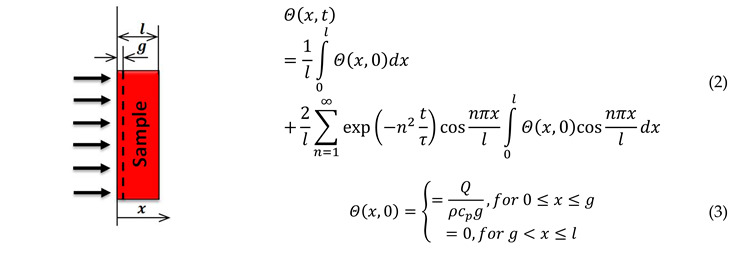


Then, the temperature difference between opposite surfaces of the sample is given by:

(4) $$\begin{array}{l} \Delta \Theta \left(t\right)=\Theta \left(0,t\right)-\Theta \left(l,t\right)=\frac{4}{l}\sum_{n=1}^{\infty }\exp\left[-{\left(2n-1\right)}^{2}\frac{t}{\tau }\right]\int_{0}^{l}\Theta \left(x,0\right)\cos\frac{\left(2n-1\right)\pi x}{l}dx\\ =4\Theta_{\infty }\sum_{n=1}^{\infty }\exp\left[-{\left(2n-1\right)}^{2}\frac{t}{\tau }\right] \end{array}$$

where $\tau =l^{2}/\left(\pi^{2}a\right)$ is the characteristic time; $\Theta_{\infty }=Q/\left(\rho c_{P}l\right)$ is the temperature increase of the sample after the end of the equalization of the heat-exchange process; *ρ* is the density; $c_{P}$ is the specific heat of the sample.

**Figure 1:**
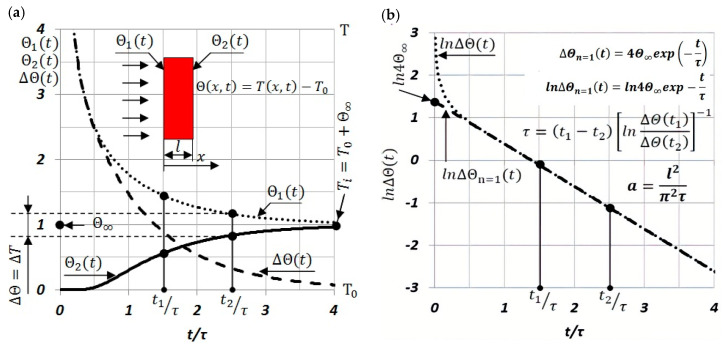
Rule for determining the thermal diffusivity of a sample using modified-pulse method (MPM). (**a**) Temperature changes on opposite surfaces of the sample; (**b**) procedure for determining characteristic time and thermal diffusivity.

Temperature changes on the front $\Theta_{1}\left(t\right)=\Theta \left(0,t\right)$*,* and rear $\Theta_{2}\left(t\right)=\Theta \left(l,t\right)$, surfaces of the test sample, and the difference ΔΘ(t) of these temperatures are illustrated in Figure 1a. Figure 1b shows how to determine characteristic time τ and thermal diffusivity a(T) of the test sample.

After truncation of the infinite series in Equation (4) for n>1, we obtain:

(5) $$\Delta \Theta \left(t\right)=4\Theta_{\infty }\exp\left(-t/\tau \right)$$

The error of this operation was below 1% if *t*/*τ* > 0.58.

In order to determine thermal diffusivity (*a*) on the basis of records of the relevant part of temperature difference Δ*Θ*′(*t*), characteristic time *τ* should first be designated. From Equation (5), we obtain:

(6) $$y=\ln\;\Delta \Theta^{\prime }\left(t\right)=\ln\left(4\Theta_{\infty }\right)-t/\tau$$

Then, data after logarithmic transformation were approximated in a specified range [*t*_1_*, t*_2_]. Least-squares approximation was used. Parameters *Θ*_∞_ and *τ* were derived from a linear approximation. On the basis of characteristic time *τ* and sample thickness *l*, thermal diffusivity *a* is calculated from:

(7) $$a=l^{2}/\left(\pi^{2}\tau \right)$$

Thermal diffusivity determines the theoretical relationship Δ*Θ*(*t*) (see Equation (4)). On the basis of this curve, we calculated the square root of the sum of squares of differences between the recorded and the theoretical value of the temperature difference between two opposite sides of sample Δ*Θ*(*t*). This parameter is minimized with respect to *t*_1_, *t*_2_, and signal shift Δ*Θ*′(*t*) in such a manner that the whole procedure is repeated with changed values of these parameters. The process is stopped when satisfactory compliance between the theoretical curve Δ*Θ*(*t*) and recorded experimental one Δ*Θ*′(*t*) is achieved.

**Figure 2:**
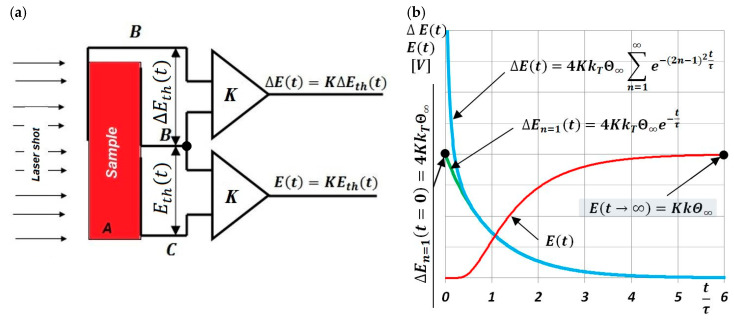
(**a**) Block diagram of system for the measurement of thermoelectric voltages $\Delta E_{th}\left(t\right)$ and $E_{th}\left(t\right)$; (**b**) typical voltage changes ΔE(t). and E(t) as a function of dimensionless time.

Measuring signals $\Delta \Theta^{\prime }\left(t\right)$ and $\Theta_{2}^{\prime }\left(t\right)$, which were used to determine the discrete thermal-diffusivity values $a\left(T_{i}\right)$ of the tested sample at $T_{i}$ temperature, were recorded using thermocouples, as shown in Figure 2a, in the form of thermoelectric voltages $\Delta E\left(t\right)=K\Delta E_{th}\left(t\right)=Kk_{T}\Delta \Theta^{\prime }\left(t\right)$ and $E\left(t\right)=KE_{th}\left(t\right)=Kk\Theta_{2}^{\prime }\left(t\right)$. The procedure for determining the typically unknown Seebeck coefficient $k_{T}\left(T\right)$, according to [39,42], is given by:

(8) $$k_{T}=0.25k\frac{\Delta E_{n=1}\left(t=0\right)}{E\left(t\to \infty \right)}$$

where $k_{T}\left(T\right)$, Seebeck coefficient of the junctions “material of the sample—thermoelectric wire”; and k, Seebeck coefficient of the junction “thermoelectric wire B—thermoelectric wire C”, could be obtained from, e.g., [44].

Temperature value $T_{i}$ in which the thermal diffusivity $a\left(T_{i}\right)$ is determined is $T_{i}=T_{0}+\Theta_{\infty }$.

The value of $a\left(T_{i}\right)$ is its average value in temperature range $T_{i}\pm 0.5\;\Delta T$ (see Figure 1a):

(9) $$a\left(T_{i}\right)={a\left(T_{i}\right)|}_{T_{i}-0.5\Delta T}^{T_{i}+0.5\Delta T}$$

In this case, the temperature-averaging range Δ*T* of thermal diffusivity $a\left(T_{i}\right)$, is equal to:

(10) $$\Delta T=4\Theta_{\infty }\exp\left[-t_{2}/\tau \right]$$

## 3. Sample and Its Investigation Preparation 

Data on the chemical composition, structure, dimensions, and density of the tested sample of the Fe–Ni alloy with the Fe65Ni35 symbol are presented in Table 1.

**Figure 3:**
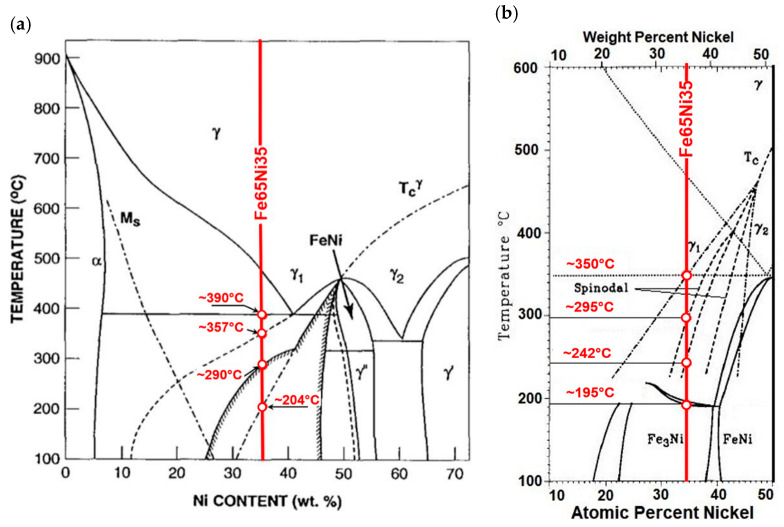
Investigated Fe65Ni35 alloy. (**a**) Fe–Ni phase diagram proposed by Reuter et al. [22,23] and Yang et al. [25], and (**b**) part of the comparison of cluster-variation and spinodal calculations with the equilibrium boundaries proposed by Chuang et al. [21] and Swartzendruber [24].

Its location was marked on phase-equilibrium diagrams of iron–nickel alloys, shown in Figure 3. The phase-equilibrium diagram by Reuter et al. [22,23] and Yang et al. [25], presented in Figure 3a, considers both the results of meteorites and alloys exposed to the electron beam, and the results of theoretical calculations made by Chuang et al. [21].

The justification for the adoption of the phase diagram shown in Figure 3a as the basis for the interpretation of experiment results was:

- during the measuring cycle, the sample face was not shielded from the direct interaction of laser-radiation quanta (without oxides or another coating);
- the interaction of the photons of the laser pulse with atoms and free electrons in the surface layer of the examined sample caused heat fluxes below the surface, which were created separately by these two carriers;
- each time the laser pulse impacted on the material of the tested sample, it positively influenced the ordering of the sample’s structure.

Measurements of temperature $T_{0}^{\prime }$ and temperature changes of $\Delta \Theta^{\prime }\left(t\right)$ and $\Theta_{2}^{\prime }$(*t*) were carried out by means of thermocouples that were electrically welded to the opposite surfaces of the investigated samples, as shown in Figure 2a. In order to measure thermoelectric-voltage difference ∆*E_th_*(*t*), i.e., to measure temperature difference ∆*Θ*′(*t*), pairs of CuNi or Fe thermocouple wires were used ((Figure 2a)—thermocouple wire B was attached to the extreme surfaces of the sample). However, to measure thermoelectric voltage *E_th_*(*t*), and thus temperature *Θ*′_2_(*t*), only on the back surface of the sample, the Fe–CuNi thermocouple (see Figure 2a—thermocouple wires B and C) was attached only to the back side of the sample. It was assumed that the temperature range of the tests would be between ambient temperature to approximately 500 °C, and the full measurement cycle would include both heating and cooling of the examined sample. Additionally, time interval Δt between subsequent discrete measurements *a*(*T_i_*) and *a*(*T*_(*i*+1)_), required for changing the temperature of the examined sample from *T_i_* to *T*_(*i*+1)_, must be experimentally determined each time—observing the dynamics of temperature changes in the sample-thermostating *T*_(0,*i*)_—after setting a new value of *T*_(0,*i*+1)_ to the automatic power-supply heater in the vacuum furnace. The next measurement ∆*Θ*(*T*_(*i*+1)_), and thus *a*(*T*_(*i*+1)_), was taken when changes d*T*_(0*,i*+1)_/d*t* reached a small previously assumed value. In turn, the selection of values Δ*T*_0_ = *T*_(0,*i*+1)_ − *T*_(0,*i*)_, i.e., between successive temperature values in thermostating, an examined sample depends heavily on changes in thermal diffusivity d*a*(*T*)/d*T* of the sample material in the function of temperature. The larger the changes of this derivative are, the smaller the value ∆*T*_0_ that must be selected so as to not overlook the initial or final value of the phase-transformation point.

## 4. Results

**Figure 4:**
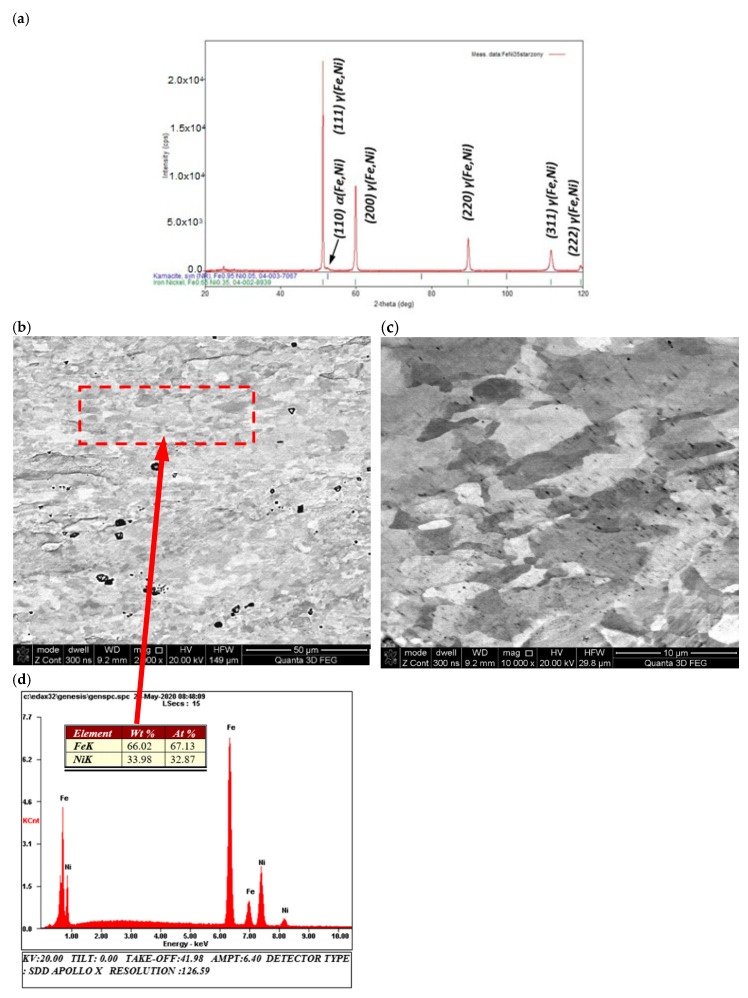
(**a**) X-ray diffraction (XRD) patterns of the austenitic γ (Fe, Ni) with trace amounts of ferrite α (Fe, Ni) structure of the Fe65Ni35 sample (**b**) before thermal-diffusivity tests characterized by (SEM/BSE) (**c**) homogeneous morphology of fine-grained structure (SEM/BSE), and (**d**) microanalysis results of chemical composition from area marked in (**b**).

**Figure 5:**
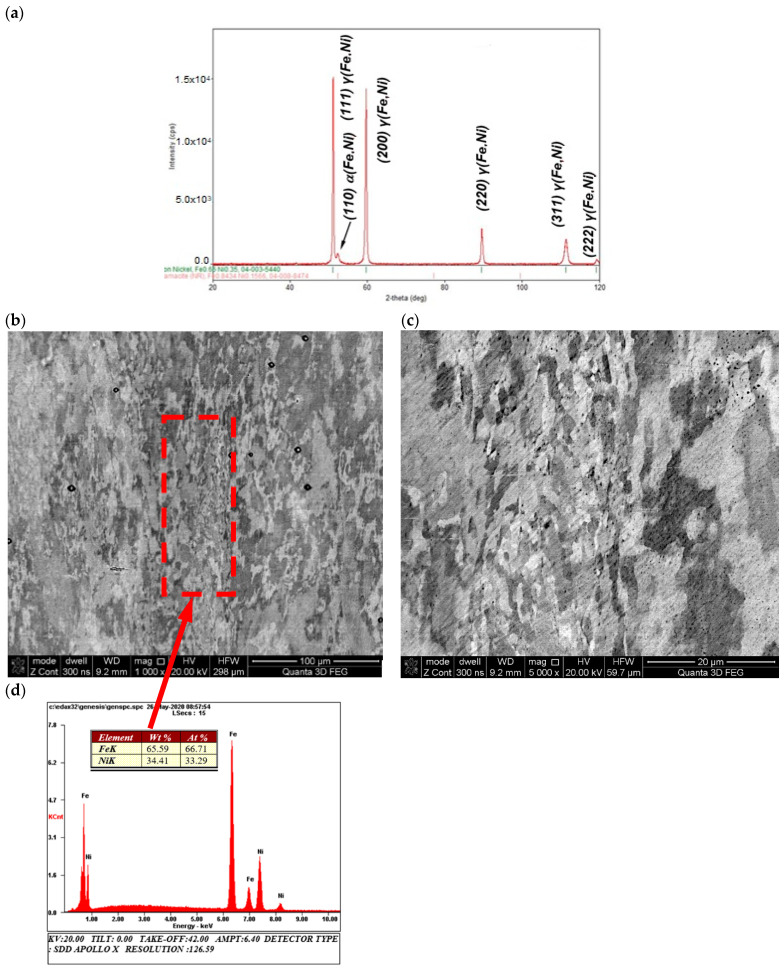
XRD patterns of (**a**) austenitic γ (Fe, Ni) with trace amounts of ferrite α (Fe, Ni) structure of Fe65Ni35 sample (**b**) after supersaturation from 340 °C (SEM/BSE) with (**c**) visible bimodal morphology of fine-grained structure (SEM/BSE) and (**d**) microanalysis results of chemical composition from area marked in (**b**).

The structural tests, in particular microscopic observations of scanning electron microscopy (SEM), supported by the microanalysis of the chemical composition of energy-dispersive X-ray spectroscopy (EDS) and the X-ray diffraction phase analysis, of the material carried out before the thermal-diffusivity tests showed, for the entire alloy, a homogeneous, fine-grained morphology of the granular structure (Figure 4b,c) of nickel austenite γ (Fe, Ni) with trace amounts of ferrite α (Fe, Ni) (Figure 5a). Supersaturation was observed above 340 °C, i.e., above the eutectoid temperature no further visible differences in the phase structure of the material were observed, as shown by XRD analysis (Figure 5a). However, it was possible to observe two grain size populations, fine and coarse (Figure 5b,c), but both with the same chemical composition.

This observation suggested that the alloy’s structure may form areas with different thermal stability related to the spinodal decomposition or the formation of metastable phases [21,22,23,24,25,26,31]. Nevertheless, in order to uniquely identify the phase changes taking place by means of classical metallurgical techniques, it is necessary to use high-resolution transmission electron microscopy (HRTEM) diffraction or to conduct electron-backscatter-diffraction (EBSD) testing.

**Figure 6:**
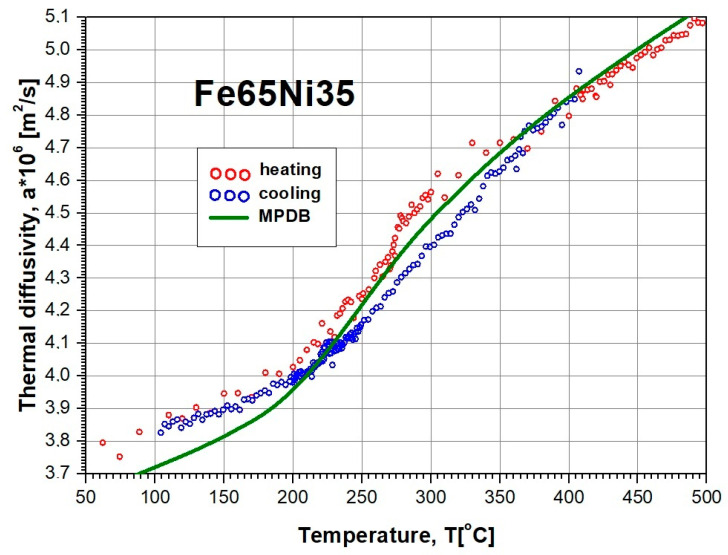
Results of own investigation of thermal diffusivity $a\left(T_{i}\right)$ for Fe65Ni35 alloy during sample heating and cooling, and results obtained indirectly for Fe64Ni36 alloy using dependence of $a=\lambda /\left(\rho c_{P}\right)$ and data from MPDB database [45].

The results of tests of thermal-diffusivity $a\left(T_{i}\right)$ changes of the Fe65Ni35 alloy in the temperature range from room temperature to approximately 500 °C are shown in Figure 6. The qualitative nature of the thermal-diffusivity changes was comparable with results obtained indirectly for the Fe64Ni36 alloy, using the dependence of $a=\lambda /\left(\rho c_{P}\right)$ (*a* is a thermal diffusivity, *λ* is a thermal conductivity, *ρ* is density, *c_p_* is specific heat) and data from the MPDB database [45].

**Figure 7:**
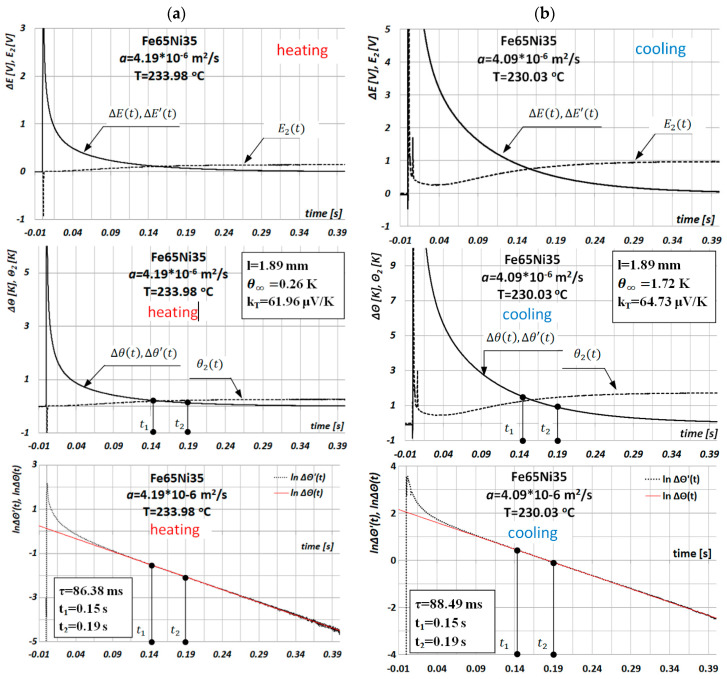
Exemplary results of discrete tests as a function of temperature of thermal-diffusivity values $a\left(T_{i}\right)$ of Fe65Ni35 alloy by the MPM (**a**) during heating $T_{i}=233.98\;℃$ and (**b**) during cooling $T_{i}=230.03\;℃$.

The results of discrete tests as a function of temperature of thermal-diffusivity values $a\left(T_{i}\right)$ of Fe65Ni35 alloy by the MPM during the heating and cooling of the sample are shown in Figure 7.

The experiment was carried out for the sample during one test cycle, which consisted of results $a\left(T_{i}\right)$ obtained in the course of heating and cooling in the temperature range taken for the tests. During sample heating, the time interval $\Delta t=t_{i+1}-t_{i}$ between consecutive discrete measurements $\Delta \Theta^{\prime }\left(T_{i}\right)$ was 20 min. During sample cooling, the time interval Δt was determined by the time constant of the heat exchange between the sample and heating element of the vacuum furnace, and was above 20 min.

**Figure 8:**
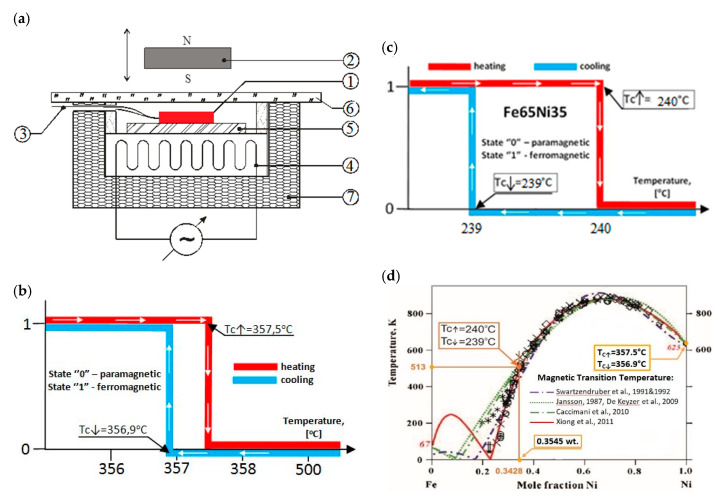
(**a**) Test bench for Curie point of Fe–Ni alloy investigation. ① Investigated sample, ② magnet, ③ thermocouple, ④ heater, ⑤ Cu plate (1.3 mm thick), ⑥ glass (1 mm thick), ⑦ insulation. (**b**,**c**) Preliminary results of Curie point investigation of suitable Ni999 and Fe65Ni35 alloys during sample heating and cooling; (**d**) graph fragment with results of experimental studies and theoretical calculations of the face-centered cubic (FCC) phase transformation of Fe–Ni alloy ([27], Figure 1), with results of $T_{C↑}$ and $T_{C↓}$ of Fe65Ni35 and Ni999 alloys (ferromagnetic 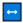 paramagnetic).

Similarly, as in [39,40], in this case, the sample of Fe65Ni35 was subjected to an examination using an approximate-value external magnetic-field to identify changes in its structure as a function of temperature (ferromagnetic 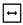 paramagnetic). The test was carried out on an identical stand as that in [39] during one cycle, including the heating and cooling of the tested sample. Test-bench testing was carried out on a Ni999 sample with a diameter of 12 mm and a thickness of 1.85 mm. The layout of the test bench and the results of these tests are shown in Figure 8.

Comparing the Tc measurement results of the tested samples using an external magnetic field with the results of other researchers available in the literature, we obtained very narrow temperature hysteresis loops for the magnetic transformations in the case of the Ni999 ($T_{C↑}=357.5\;℃$ and $T_{C↓}=356.9\;℃$) and Fe65Ni35 ($T_{C↑}=240\;℃$ and $T_{C↓}=239\;℃$) alloys. According to [24] and Figure 8, for Ni, $T_{C}=354.3-360\;℃$, and for FeNi at 35.3 at % Ni, $T_{C}=228-261\;{}^{\circ}C$.

**Figure 9:**
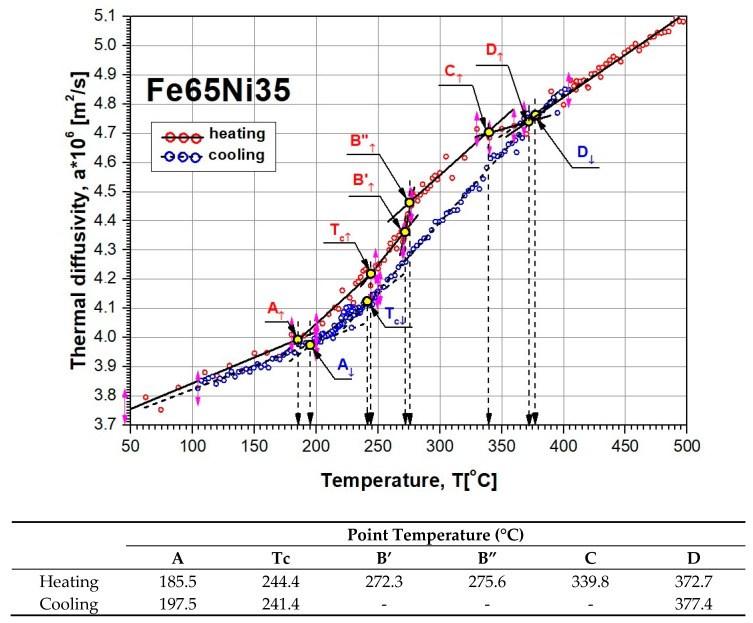
Temperature characteristics of thermal-diffusivity a(T) changes of Fe65Ni35 adopted for further analysis together with estimated values of its characteristic points.

**Figure 10:**
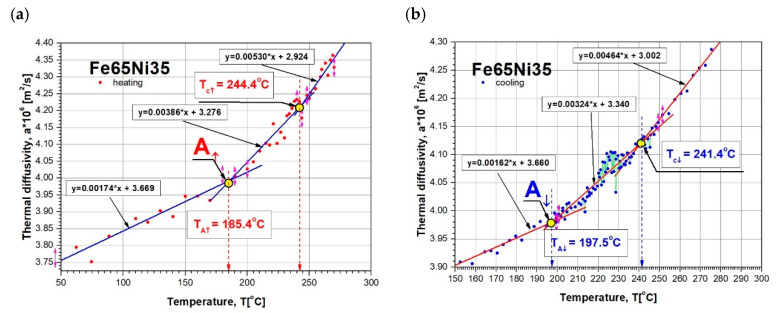
Example of $T_{C}$ temperature determination as the point of linear-regression-line intersection of Fe65Ni35 thermal-diffusivity changes during (**a**) heating and (**b**) cooling.

After the initial elaboration of discrete results of our own tests of thermal diffusivity $a\left(T_{i}\right)$, shown in Figure 6 (using fragments of linear approximation), characteristic temperatures (A, B, … D) of the changes in a(T) were determined for sample heating and cooling. Values A, B, … D, in which da(T)/dT changes occurred, responsible for phase transformations and magnetic transformation, were determined from the intersection of simple linear regressions that were used to approximate the experimentally obtained values of thermal diffusivity. The values of a(T), obtained in this way with the temperature values given in the table at characteristic points, are shown in Figure 9. The determination of the temperature value of magnetic transitions for during heating $T_{c↑}$ and cooling $T_{c↓}$ of the tested sample are shown in Figure 10.

## 5. Discussion

**Figure 11:**
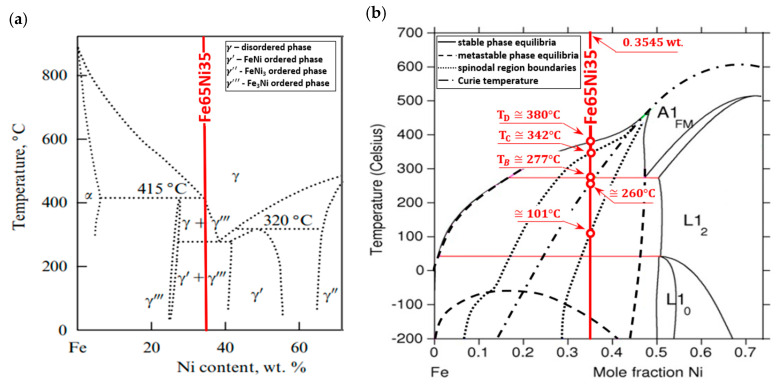
Investigated Fe65Ni35 alloy marked on fragments of Fe–Ni phase diagrams proposed by (**a**) Chamberod et al. [31], and (**b**) Cacciamani et al. [26], covering only phases based on a FCC crystal structure.

The Fe–Ni alloy selected for testing was an alloy with the formula Fe65Ni35 whose changes in thermal diffusivity a(T) were experimentally determined in one measurement cycle during sample heating (from about 50 to 500 °C) and cooling (from approximately 400 to 100 °C). The characteristics of a(T) of this alloy, together with discrete temperature values at points (A, B, C, …) where there were rapid changes in da(T)/dT derivatives, which resulted in phase transformations and magnetic transformation, are shown in Figure 9. Preliminary analysis of the effects associated with the changes of a(T) shows that they were dominant during the heating of the examined alloy, Fe65Ni35. However, the determined characteristic temperatures of changes in da(T)/dT (Figure 7), compared with the transformation temperatures described in the Fe–Ni equilibrium systems, listed in Figure 3, are completely different. This led the authors to conduct a deeper analysis of the phase equilibrium, especially in the low-temperature area below 400 °C. Excellent and very thorough analysis of this issue was made by Zemtsowa in [30], which compared the results of the investigations within the last 50 years concerning stable, metastable, and spinodal areas of the Curie temperature, and the extent of the occurrence of superstructures L10 and L12 for specific proportions of iron and nickel. In conclusion, it is recommended to use the Fe–Ni phase system as it is the most credible one, supported by experimental data proposed by Chamberod et al. [31], and shown in Figure 11a. However, the findings obtained in this work with regard to the designated temperature characteristics were not in agreement with the proposed phase diagram.

Another approach to clarify and validate the phase diagram of the Fe–Ni system is the use of thermodynamic calculations. Connecting thermodynamic modeling with experiment data, most importantly with atomistic calculations (making it possible to designate the thermodynamic functions of metastable states) presented by Cacciamani et al. in [26], the Fe–Ni equilibrium diagram covering phases based on the FCC crystal structure was proposed—split into an area of stable, metastable equilibrium phases, spinodal region, and Curie temperature (Figure 11b).

Considering previously published data, and comparing them with registered values of thermal diffusivity based on the heating curve (Figure 9), it could be observed that the obtained experimental results of temperature changes were most similar to the equilibrium system, presented in Figure 11b. The temperature of the eutectoid transformation γ-(Fe,Ni)_FM_ → α-Fe + FeNi3 (Point B, Figure 9) was occurred the temperature interval 272.3–275.6 °C, close to 277 °C, as shown in Figure 11b. The eutectoid temperature had a constant value. The presented experimental data were included in a range of approximately 3 K. This effect was undoubtedly linked with the registration of thermal diffusivity at a slow yet constant temperature change of 0.5 K/min. Very high convergence was also obtained in the case of the temperature recorded at Point C (339.8 °C) that, on a comparable equilibrium diagram, corresponded to 342 °C, in which the spinodal area of phases γ-(Fe,Ni)_PM_ + γ-(Fe,Ni)_FM_ lost its domain arrangement and was being rebuilt in a metastable area of solubility variable γ-(Fe,Ni)_PM_ + γ-(Fe,Ni)_FM_. At 372.7 °C (Point D, Figure 9) and 380 °C, in accordance with Figure 11b, there was a monotectoid transformation of a mixture of γ-(Fe,Ni)_PM_ + γ-(Fe,Ni)_FM_ into the solid solution of γ-(Fe,Ni)_FM_.

However, the Curie temperature $T_{C}$, both during cooling (241.4 °C) and heating (244.4 °C), although clearly differing from the value shown in Figure 11b (~260 °C), was comparable with data shown in Figure 8, hence acknowledging and validating the possibility of applying the research technique to analyze phase transformations. The observed difference $\Delta T_{C}$ of approximately 20 °C may be explained on the basis of research results by M.R. Gallas and J.A.H. da Jornada published in [33]. They demonstrated that the observed effect may be caused by changes in the mutual interactions of Fe–Fe and Fe–Ni in areas of the solubility variable and spinodal decomposition, which affect diffusion changes. Curie temperature changes may also be due to slight structural changes already occurring at a crystallite level deriving from material purity and the rate of temperature changes. The significant differences in the designation of $T_{C}$ values in Fe–Ni alloys were also discussed in [30]. The authors observed that, for the same alloy of comparable and very high purity, achieved by melting and vacuum casting of high-purity components, despite the application of the same research techniques in two different cases $T_{C}$, was first registered at 415 and then at 270 °C. The authors claim that the observed effect may have been caused by the presence of interstitial elements in the alloy, e.g., at a carbon content of 0.001%, and temperature, $T_{C},$ equal to 470 °C, while increasing the carbon content to 1.7% resulted in a rise in $T_{C}$ of up to 580 °C.

The most difficult feature to interpret seems to be the temperature recorded at Point A. On the basis of analysis carried out in the available literature, the authors postulate that, at this temperature, in accordance with Figure 3b, the rebuilding of a metastable area in the solubility variable γ-(Fe,Ni)_PM_ + γ-(Fe,Ni)_FM_ into a superstructure L1_2_ of phase Fe_3_Ni occurs. However, this should be confirmed by structural research, which is not the subject of this work.

Clarification is also required with regard to a thermal diffusivity hysteresis loop of the examined Fe65Ni35 alloy, observed between the heating and cooling of the sample. However, this effect is presumably linked with the lack of transformations registered during cooling, which is undoubtedly connected with the phenomenon of overcooling. The high thermal stability of this material, causing stable structures compatible with the equilibrium system only at cooling speeds of approximately 1K/(10^6^ years) [22], results in overcooling when cooling at a speed of 0.5 K/min of the γ-(Fe,Ni)_PM_ of the A1 lattice, up to the temperature of approximately 200 °C, in which an intermetallic phase Fe_3_Ni of the L1_2_ structure is formed.

## 6. Conclusions

The application of the modified-pulse method to identify phase transformation in the case of the Fe65Ni35 alloy during heating confirmed the theoretical results reported in the phase diagram by Ciaciamani et al. [26]

This allows the use of the MPM to identify and verify phase changes occurring in the tested alloys on the basis of changes in their thermal-diffusivity characteristics, *a*(*T*). This can be used as an additional research tool to identify and verify tests carried out by other methods, and in some cases, it can be an important aid in analyzing phase transformations occurring at the domain level that are difficult to identify by classical metallurgical methods, or in identifying order–disorder or/and magnetic transformation.

## Figures and Tables

**Table 1:** Basic data of examined sample.

Alloy	D/l (mm)	Density (kg/m^3^)	Content, (wt %)									Sample Phase Structure before Experiment (%)	
			Ni	Fe	Co	Cu	Cr	Al	Zn	Si	Ca	α	γ
Fe65Ni35	12.0/1.89	8030 ± 130	35.45	bal.	0.05	0.1	0.01	0.005	0.005	0.005	0.005	5.69	94.31
